# Novel frameshift mutation in the insulin (*INS*) gene in a family with maturity onset diabetes of the young (MODY)

**DOI:** 10.1111/1753-0407.12849

**Published:** 2018-10-17

**Authors:** Xiaoyu Xiao, Lili Liu, Yang Xiao, Zhiguo Xie, Long Li, Houde Zhou, Weili Tang, Shiping Liu, Zhiguang Zhou

**Affiliations:** ^1^ Department of Metabolism & Endocrinology, The Second Xiangya Hospital Central South University Changsha Hunan China; ^2^ Key Laboratory of Diabetes Immunology (Central South University) Ministry of Education, National Clinical Research Center for Metabolic Diseases Changsha Hunan China

**Keywords:** diabetes, insulin gene, MODY, mutation

## Abstract

**Highlights**

- This study reports a novel frameshift mutation c.212dupG (p.Gly73fs) in the insulin (*INS*) gene causing maturity onset diabetes of the young (MODY) 10, one of the rare types of MODY, identified in seven family members.
- Screening for mutations in identified MODY genes is warranted in patients who require insulin, are negative for autoantibodies but have a family history of diabetes.

## Introduction

**Figure 1:**
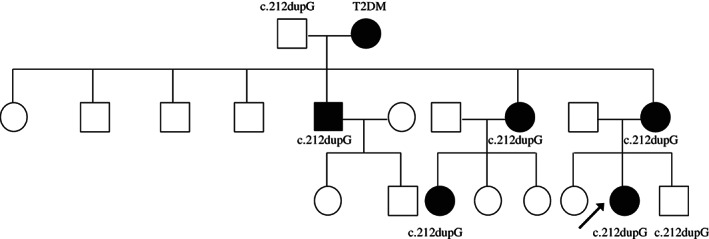
The family pedigree. The arrow indicates the proband. Squares represent males and circles represent females, diabetic people are indicated by filled symbols. Sanger sequencing revealed a novel mutation in the insulin (*INS*) gene c.212dupG present in the family members as indicated in the pedigree. The *INS* mutation was inherited from the proband's grandfather. T2DM, type 2 diabetes mellitus

Maturity onset diabetes of the young (MODY) accounts for 1‐2% of all diabetes. It is caused by autosomal dominant mutations, and 14 genetic MODY subtypes have been identified to date.^1^ One of the rare types of MODY, MODY10, is caused by heterozygous insulin (*INS*) gene mutations.^2^ Herein we report on a 23‐year‐old proband with familial diabetes in three generations (proband, her mother, maternal grandmother, maternal uncle, maternal aunt, and maternal female cousin). This disease most likely results from a novel frameshift mutation c.212dupG (p.Gly73fs) in the *INS* gene identified in seven family members (Figure 1).

## Case report

The proband was born in 1994 at full term with a birth weight of 3900 g from an uneventful pregnancy in which gestational diabetes screening was not performed. The proband was diagnosed with hyperglycemia in 2009 due to typical diabetic symptoms without signs of diabetic ketoacidosis and was treated with metformin and gliclazide. In 2011, she presented to the Department of Metabolism & Endocrinology, The Second Xiangya Hospital, Central South University because of blurred vision and was diagnosed with metabolic cataracts. The proband was started on insulin (before meals and at bedtime) for 1 month until her blood glucose was under control and subsequently underwent cataract surgery. Insulin therapy was then suspended. The proband started insulin therapy again in 2016 with little improvement in glucose levels due to frequently skipped injections. In 2016, she was admitted again to the Department of Metabolism & Endocrinology for uncontrolled diabetes and was diagnosed with polycystic ovary syndrome, diabetic nephropathy, and peripheral neuropathy. The patient was not insulin resistant and did not have diabetic retinopathy or macroangiopathy. Her body mass index (BMI) was 23.3 kg/m^2^, with fasting glycemia 12.02 mmol/L, 2‐hours postprandial glycemia 22.94 mmol/L, HbA1c 12.3%, fasting C‐peptide (FCP) 115.8 pmol/L (reference range 223.4‐746.2 pmol/L), and 2‐hours postprandial C‐peptide 325.1 pmol/L. The patient was negative for the islet autoantibodies glutamic acid decarboxylase antibody, insulinoma‐associated protein‐2 antibody, and zinc transporter 8 antibody. The fasting serum proinsulin concentration was 3.39 ng/mL. The patient has been treated with metformin and insulin since then.

The mother of the proband was born in 1966 and has suffered from diabetes since 2002; she also has metabolic cataracts (BMI 23.9 kg/m^2^) and is now using premixed insulin twice a day. The proband's maternal grandmother (at the time of writing aged 78 years, BMI 24.4 kg/m^2^, FCP 1194.6 pmol/L) was diagnosed with diabetes at 67 years of age and is being treated with premixed insulin once a day with metformin. Diabetes was also diagnosed in the proband's maternal uncle (at the time of writing aged 48 years, BMI 24.0 kg/m^2^, FCP 862.9 pmol/L, taking gliclazide and metformin), maternal aunt (at the time of writing aged 52 years, BMI not measured, FCP 2113.2 pmol/L, nearly blind due to diabetes and receiving dialysis, taking no medications for diabetes), and maternal female cousin (at the time of writing aged 31 years, BMI 24.4 kg/m^2^, FCP 481.5 pmol/L, taking gliclazide and metformin).

**Figure 2:**
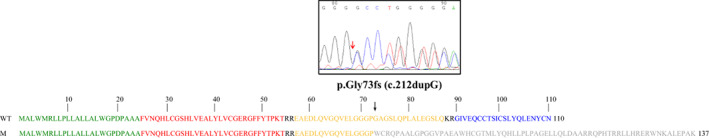
Comparison between the wild‐type (WT) molecule and the predicted mutant (M) amino acid sequence after duplication mutation at c.212dupG (demonstrated in the box), leading to a frameshift starting at amino acid residue 73 (p.Gly73fs), indicated by the black arrow. Amino acid sequences of the signal peptide are shown in green, the B‐chain is shown in red, the C‐peptide is shown in yellow, the A‐chain is shown in blue, and the mutated sequence is indicated by the gray letters. The red arrow indicates the mutation site

This study was approved by the Ethics Committee of The Second Xiangya Hospital and informed consent was obtained from the proband and her family members. The proband and available family members underwent molecular genetic testing of MODY 1‐13 genes. Sanger sequencing was used to confirm mutations identified by next‐generation sequencing. A novel heterozygous duplication mutation (c.212dupG) in the coding region, exon 3 of the *INS* gene, was detected in the proband, her younger brother, her mother, maternal grandfather, maternal uncle, maternal aunt, and maternal female cousin. This mutation leads to a frameshift starting at amino acid residue 73 of the C‐peptide region (p.Gly73fs). In silico analysis predicted the original stop codon was lost due to the frameshift mutation, resulting in a longer protein molecule containing an additional 27 amino acids (Figure 2). The variant detected was not found in polymorphism databases (i.e. the Exome Sequencing Project, 1000 Genome Project, and dbSNP databases).

## Discussion

Herein we report on an *INS* MODY family with a frameshift mutation in exon 3 of the *INS* gene c.212dupG (p.Gly73fs), which, to the best of our knowledge, has been detected for the first time. Mutations in the *INS* gene affect a variety of different steps of insulin biosynthesis, leading to distinct forms of β‐cell damage. The *INS* gene encodes a single chain precursor molecule, preproinsulin, that is post‐translationally modified to insulin in the pancreatic β‐cells. Mutations in the *INS* gene affect the structure of the preproinsulin molecule, resulting in aberrant processing of preproinsulin to proinsulin or folding of proinsulin.^3^ Insulin gene mutations may present with or without hyperinsulinemia or hyperproinsulinemia.^4^

The novel frameshift mutation c.212dupG was found in the proband, her younger brother, her mother, maternal grandfather, maternal uncle, maternal aunt, and maternal female cousin. In addition to the normoglycemic maternal grandfather (at the time of writing aged 83 years, BMI 22.2 kg/m^2^, FCP 1956.8 pmol/L) and younger brother (at the time of writing aged 9 years, BMI 16.7 kg/m^2^, FCP 1020.2 pmol/L), the remaining five members of the family had had various diabetes phenotypes since the ages of 15, 36, 45, 34 and 24 years. A wide spectrum of clinical manifestations has been reported in *INS* gene mutations even within a single family,^2^, ^5^ especially considerable differences in residual β‐cell function.^6^ Clinical heterogeneity in terms of the age at diagnosis, manifestations and therapeutic options are also evident in the present case. The proband's maternal grandmother, who did not carry the mutation, suffered hyperglycemia which should be considered as type 2 diabetes mellitus instead of MODY. Conversely, the maternal grandfather carried the mutation without overt diabetes, which is probably due to incomplete penetrance.^7^ The proband's younger brother who carries the mutation did not have diabetes at the time of this study, but may develop diabetes in the future.

Metabolic cataracts of the proband and her mother are likely secondary to hyperglycemia. Diabetes is an established risk factor for the occurrence of cataracts, although the exact etiology of cataract formation in diabetes remains unknown. Cataracts are not a classic feature of MODY or *INS* mutations. Although *INS* gene mutations have been found to be associated with acquired cataracts,^8^ there are no published data on the prevalence of cataracts in monogenic diabetes, although a MODY5 (hepatocyte nuclear factor‐1 beta [*HNF1B*] gene mutation) adolescent female was diagnosed with bilateral cataracts at the time of presentation.^9^

Based on the 2015 guidelines developed by the American College of Medical Genetics and Genomics (ACMG) for the classification of pathogenic or likely pathogenic variants,^10^ the novel *INS* mutation identified in this family was considered to be “pathogenic”, based on the evidence framework of PVS1 (pathogenic very strong; i.e. a null variant [i.e. frameshift]), PM2 (pathogenic moderate; i.e. not found in polymorphism databases), and PP4 (pathogenic supporting; i.e. the patient's phenotype and family history highly specific for MODY). However, more stringent criteria and evidence are necessary to establish pathogenicity. Screening for mutations in identified MODY genes is warranted in patients who require insulin, are negative for autoantibodies but have a family history of diabetes.

## Disclosure

None declared.
